# Foxp3 Instability Helps tTregs Distinguish Self and Non-self

**DOI:** 10.3389/fimmu.2019.02226

**Published:** 2019-09-24

**Authors:** Zhongmei Zhang, Xuyu Zhou

**Affiliations:** ^1^Experimental Immunology Branch, National Cancer Institute, National Institutes of Health, Bethesda, MD, United States; ^2^CAS Key Laboratory of Pathogenic Microbiology and Immunology, Institute of Microbiology, Chinese Academy of Sciences (CAS), Beijing, China; ^3^Savaid Medical School, University of Chinese Academy of Sciences, Beijing, China

**Keywords:** Treg, Foxp3, instability, self and non-self-discrimination, TCR

## Abstract

Regulatory T cells (Tregs) are small subsets of CD4 T cells that play a central role in the controlling of immune tolerance. Tregs are either generated in the thymus (tTregs) or the periphery (pTregs), and both express the master transcription factor Foxp3. Stable expression of Foxp3 is important for the maintenance of Tregs identity and their suppressive function. Similar to conventional T cells, Tregs can recognize both self- and non-self-antigens, and TCR engagement leads to Treg activation and the generation of effector Tregs. Emerging shreds of evidence suggest Tregs are not always stable, even fully committed mature tTregs, and can lose foxp3 expression and programming to effector-like T cells. In this review, we summarize recent findings in Treg instability and the intrinsic and extrinsic mechanisms in controlling the Foxp3 expression. Finally, we propose a new hypothesis that Foxp3 instability might help tTregs distinguish between self and non-self-antigens.

## Introduction

T cells are one of the major components of the adaptive immune system which protects against all kind of pathogens, harmful substances, and foreign tissues. Immature T cells expressing an enormous number of TCRs generated by random VDJ recombination undergo selection in the thymus, where self-reactive T cells are clonally deleted through negative selection. However, this mechanism of controlling self-reactive T cells, known as central tolerance, is not perfect. Some potentially autoreactive T cells escape deletion in the thymus and migrate to the periphery. In recent years, we have learned that suppression of autoreactive lymphocytes relies on a subset of T lymphocytes called regulatory T cells, a small subpopulation of CD4^+^ T cells characterized by expression of the forkhead transcription factor Foxp3 (1).

Nishizuka and Sakakura were the first to show that thymus-derived Tregs mediate dominant self-tolerance (2). Their study showed that neonatal thymectomy around day 3 after birth resulted in severe autoimmune diseases, which could be prevented by adoptive transfer of thymocytes or splenocytes from adult euthymic mice (2). These observations demonstrated that a T cell subset generated in the mouse thymus after the third day of life could prevent autoimmunity. This T cell subset was later identified as a thymus-derived CD25^+^ CD4^+^ T cell population capable of protecting animals from autoimmune diseases (3, 4). However, CD25 is an activation marker, and by itself, is insufficient for identifying tolerance- and inflammation-promoting cells during an immune response. The breakthrough in the field came when the transcription factor Foxp3 was identified as the master regulator of Tregs (5–7). Expression of Foxp3 faithfully distinguishes naturally occurring thymic, as well as peripheral, CD25^+^CD4^+^ Tregs from naive CD25^−^CD4^+^ T cells or activated CD4^+^ T cells. Moreover, sustained Foxp3 expression in mature Tregs is critical for maintaining of the Treg cell identity and suppression of life-threatening autoimmunity (8).

Although Tregs are likely to represent a stable cell lineage with regulatory functions, accumulating evidence suggests that Foxp3^+^ Tregs retain plasticity and can be “reprogrammed” into T helper cells under certain environmental conditions (9–12). In this review, we summarize recent results on Treg instability and discuss their implications in distinguishing self and non-self.

## Mechanisms of Foxp3 Induction and Maintenance

The induction and maintenance of Foxp3 distinguish Tregs from the other T helper cell populations. Interestingly, these two processes are largely separable and regulated by Foxp3 promoter and three conserved non-coding sequences (CNS) located around the first intron and the first coding exon of the Foxp3 gene (13–15). Intrathymic differentiation of Tregs is synchronized with positive and/or negative selection and starts mostly at the CD4^+^CD8^−^ single-positive (CD4SP) stage (16, 17). By using TCR and antigen double transgenic systems, it was shown that CD25^+^CD4^+^ cells can differentiate into Tregs in the thymus when the cognate antigen is presented by thymic stromal cells (18, 19). These results suggest that Treg cells develop from CD4 SP T cells possessing TCRs with high avidity toward self-antigens (20, 21). Thymic Foxp3 induction and Treg lineage commitment are the synergistic effects of TCR signaling, co-stimulatory signals through CD28 and common γ-chain cytokine signals, particularly IL-2 signal (22–26). Transcription factors such as NFAT, AP-1, Nr4a factors, and STAT-5 can drive Foxp3 promoter activation in response to TCR and IL-2 signaling (27–29). Tregs can also be induced in the periphery from naïve conventional CD4^+^ T cells, and these pTregs play an important role in maintaining intestinal mucosal immune tolerance and maternal-fetal tolerance (30–32). Interestingly, pTregs and tTregs have different dependence on CNS-1, which contains a TGF-β-NFAT response element, and is indispensable for peripheral, but not thymic, Foxp3 induction (15, 33). Additionally, CNS3, another cis-element regulating Foxp3 locus, acts as a pioneering element essential for inducing of Foxp3 expression (13). CNS3 recruits c-Rel and Foxo family transcription factors such as Foxo1 and Foxo3, which can open the Foxp3 gene locus, thereby facilitating Foxp3 induction (34, 35).

Stable expression of Foxp3 is dependent on CNS2, a CpG-rich region within Foxp3 locus. CNS2, also called Treg specific demethylation region (TSDR), is dispensable for Foxp3 induction but essential for heritable maintenance of Foxp3 expression in dividing Tregs (13, 14). CNS2 is fully methylated in conventional T cells (Tconvs) and highly methylated in Tregs generated recently in the thymus. Demethylation of CNS2 contributes to stable Foxp3 expression in Tregs (36, 37). The initiation of TSDR demethylation is Foxp3-independent, as the “wannabe” Tregs, which transcribe the Foxp3 locus but do not express Foxp3 protein, show demethylation of the TSDR (13, 36). Recent studies have revealed the roles of Ten-eleven translocation (TET) proteins, which can induce the demethylation of 5-methylcytosine (5mC) in a cell cycle-independent way, in the demethylation of CNS2 (38). By using CD4-Cre and Foxp3-Cre-mediated depletion of Tet2/Tet3, Rao et al. and Yoshimura et al. showed that the Tet proteins play a critical role in demethylating TSDR to ensure stable Foxp3 transcription (39, 40). Once the TSDR has been demethylated, Foxp3 protein, cooperating with other transcription factors such as Runx/Cbfβ, STAT5, CREB/ATF, and Ets-1, binds to the demethylated TSDR and stabilizes its own transcription through a positive feedback mechanism (13, 41–43). Rao et al. also observed that four CpGs in CNS1 and 11 CpGs in CNS2 share a similar CpG methylation pattern (38). Collectively, these findings show that the establishment of stable expression of Foxp3 occurs in a two-step process, the first step being the Tet- dependent demethylation of TSDR, followed by Foxp3-dependent self-enforcement. Moreover, the metabolic status during tTreg cell activation and environmental cytokine cues also contribute to the stability of Foxp3 expression (44–49).

## Evidence of Instability of Foxp3

Accumulating evidence suggests that Foxp3^+^ cells are not terminally differentiated. Indeed, *in vitro* culture system and *in vivo* transfer experiments showed that a fraction of Tregs can lose their Foxp3 expression and acquire the ability to produce the corresponding Th cytokines depending on their microenvironment (10, 50, 51). To overcome the limitations of the relatively artificial experimental setting used in these experiments and directly address the problem *in vivo*, we have used genetic lineage-tracing approaches. To identify exFoxp3 T cells in the normal T cell repertoire, we generated a fate-mapping system by crossing ROSA26 YFP-reporter mice with Foxp3 bacterial artificial chromosome (BAC) transgenic mice expressing a GFP-Cre fusion protein (9). We found that 10–15% of YFP^+^ cells did not express Foxp3 and GFP, and these GFP^−^YFP^+^ cells displayed an activated memory T cell phenotype with the ability to produce pro-inflammatory cytokines, IFN-γ and IL-17. Moreover, when crossed with transgenic mice expressing a diabetogenic TCR, the frequency of exFoxp3 cells increased in the inflamed pancreas and these cells conferred autoimmune diabetes upon adoptive transfer into lymphopenic mice. Similar results were observed by using MOG tetramer to identify antigen-specific Tregs in an experimental autoimmune encephalomyelitis (EAE) model, further supporting the notion that Tregs can be converted into pathogenic T helper cells *in vivo* (11). Collectively, these observations suggest that Foxp3^+^ Tregs can lose Foxp3 expression and undergo lineage reprogramming in response to certain extrinsic cues such as lymphopenia and inflammation.

## Contradiction and Possible Explanations

Conclusions drawn from these studies have generated great debates. Treg plays a critical role in maintaining self-tolerance and many Tregs are biased toward self-recognition. In this context, unlimited functional reprogramming of Tregs to pathogenic effector T cells could have a disastrous effect on the host. The reprogramming model has been challenged by a study from Rudensky et al., who utilized an inducible-labeling approach by knocking in a cDNA encoding GFP-Cre-ERT2 fusion protein into a 3′ untranslated region (UTR) of the Foxp3 gene, and then crossing these mice with ROSA26 YFP mice (52). When treated with tamoxifen to pulse label Foxp3^+^ T cells, Rudensky et al. found that <5% of YFP^+^ cells were Foxp3 negative. The frequency of Foxp3^−^ cells did not increase even under inflammatory or lymphopenic conditions, suggesting that Foxp3 expression in Tregs was remarkably stable. The contradiction is unlikely to be due to an unfaithful reflection of endogenous Foxp3 expression by Foxp3-GFP-Cre BAC construct, as an independent Foxp3 GFP-Cre knock-in x ROSA26 RFP fate mapping system also showed that about 15% of peripheral Foxp3 traced RFP^+^ T cells were indeed Foxp3^−^ (53). Interestingly, different subsets of Tregs might have different Foxp3 stability, which even changes according to their developmental stages (33, 36, 37, 54). It is well-known that *in vitro* iTregs, induced by TGF-β, have highly methylated TSDR and are prone to lose Foxp3 (37). Rudensky et al. have shown that newly generated pTregs were unstable, about 50% of lineage-traced cells were Foxp3^−^, whereas stable pTregs were generated only after 5 weeks upon transferring (33). The labeling efficiency of Tregs during fate mapping experiment, particularly the unstable pTregs, could be a potential factor for the inconsistent results among different models.

To resolve the controversies regarding the stability of Foxp3, Hori have proposed a heterogeneity model and postulated that exFoxp3s do not indicate real reprogramming of Tregs but reflect a minor population of uncommitted Foxp3^+^ T cells which have lost their Foxp3 expression (55). According to this heterogeneity model, uncommitted Tregs are a minor fraction of the Foxp3^+^CD25^−^ subset, generated either from transient Foxp3 expression during the activation of peripheral T cells, or from immature tTregs that fail to demethylate CNS2 during thymic Treg development, thereby becoming susceptible to losing Foxp3 expression in lymphopenic and *in vitro* polarization settings (50). In contrast, most, if not all, of the CD25^+^ Foxp3^+^ T cells show stable Foxp3 expression under those conditions. Although the heterogeneity model is compatible with the two fate-mapping experiments mentioned before, it fails to shed any light on whether the fully committed tTregs can lose Foxp3 expression. Moreover, CD25 expression was significantly decreased following Treg activation and functional specialization (T-bet^+^Treg, Bcl6^+^TFR, etc.) (12, 56), and effector Tregs preferentially use ICOS instead of IL-2 signaling to support homeostasis and function (57). If so, can activated/effector Tregs maintain Foxp3 expression?

## TCR Signal Determines the Instability of Treg

The heterogeneity and reprogramming models are not mutually exclusive. Recently, we have generated a new fate-mapping system which traces only the epigenetically stable tTregs, and that provides us a unique opportunity to address the above questions mentioned before (12). It has been demonstrated that the CNS1, which serves as a major TGF-β sensor, is critical for the generation of induced pTregs, but largely dispensable for tTreg development (33). Based on this observation, we developed a delta CNS1 Foxp3 BAC transgene mouse strain in which only tTregs express the Thy1.1-Cre fusion protein (referred to as Foxp3 delta CNS1-Cre-Thy1.1). Unexpectedly, the expression of Thy1.1-Cre reporter was significantly delayed in the thymus and marked the mature tTregs with hypomethylation of the TSDR. By using the Foxp3 delta CNS1-Cre-Thy1.1 x ROSA26 YFP fate-mapping system, we studied the stability of bona fide tTregs. We found that only ~1% of mature tTregs lost Foxp3 expression in secondary lymphoid organs, indicating that tTregs are stable under homeostatic conditions. However, activation and sequential functional specialization of tTregs (conversion to T-bet^+^Treg and Bcl6^+^TFR) result in the loss of Foxp3 stability and reprogramming into T helper lineage. Destabilization of Foxp3 can also happen in Th2-like Treg or Th17-like Treg. Chatila et al. have shown that selective boosting of IL-4R signaling by introducing a gain-of-function IL-4Rα (Il4raF709) in Tregs can reprogram Treg to Th2 cells (58). Similarly, augmentation of Rorγt by knocking-out both T-bet and GATA3 in Tregs results in decreasing Foxp3 expression and generation of IL-17 producing cells (59). We further demonstrated that the signal switch from IL-2 to ICOS/PI3K during Treg activation account for Foxp3 instability and Treg reprogramming (12). Initiation of TSDR re-methylation is likely the key step for loss of Foxp3 expression (9), death of activated Treg, and survival and expansion of T helper cells could further drive the conversion.

To trace the functional specification of Tregs *in vivo*, we have used a dual lineage tracing mouse model in which the genetic tracing of Foxp3 and T-bet was simultaneously enabled (12). Interestingly, “exT-bet” Foxp3^+^ cells (T-bet-tracer positive Tregs that had lost T-bet expression) reverted to resting-like Treg phenotypes with stable Foxp3 expression, whereas sustained T-bet expressing effector Tregs tend to lose Foxp3 expression. Together, these results suggest that over-stimulation likely promotes the instability of Tregs and converts them from immune-suppressing cells to immune-boosting cells.

## Foxp3 Instability May Help tTregs Distinguish Self and Non-self

**Figure 1:**
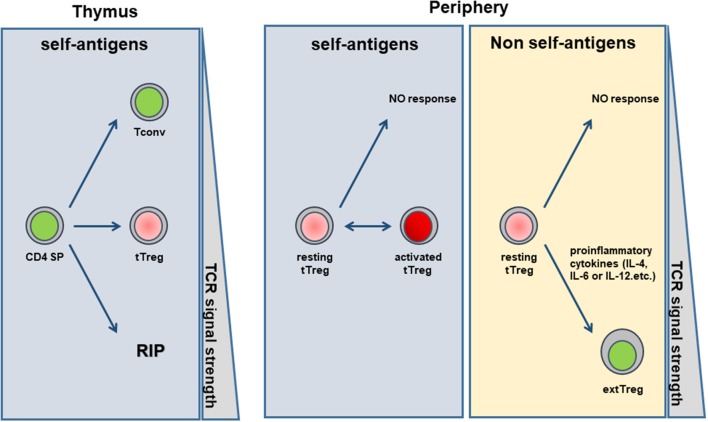
Foxp3 instability helps tTregs distinguish between self and non-self-antigens.

Considering all these observations, we propose the hypothesis that Foxp3 instability may help tTregs distinguish between self and non-self-antigens (Figure 1). Both conventional T cells and tTreg develop in the thymus, possibly from the same pool of diversified immature T cells. Thymocytes are educated by an elaborate process, during which their fate was determined by the affinity of the TCRs for self-peptide-MHC complexes on APCs. CD4 SP cells that bind with high affinity to self-antigens undergo clonal deletion to limit autoimmunity, whereas the thymocytes that bind with low affinity against the self-ligands can survive and emigrate to the periphery as conventional T cells. Tregs are positively selected from the TCRs with the affinity between the clonally deleted autoreactive T cells and Tconvs. In the periphery, Tconvs usually remain inactive due to their low affinity to self-ligands. When the organism is infected by pathogens, Tconvs, having high affinity against foreign antigens, undergo clonal expansion to differentiate into effector cells and protect the host. Similar to T conventional cells, TCR engagement is also a critical step for Treg activation and gain of potent suppressive function (60, 61). Although tTregs are selected by self-antigens in the thymus, many studies have suggested that a substantial proportion of thymic Tregs recognize foreign antigens. Because TCRs on Tregs has an intermediate affinity to self-ligands, most of the self-ligands in the periphery can only activate tTregs to a certain extent, thereby maintaining a perfect window in which Treg activation is triggered but their stability is not impaired. In contrast to self-antigens, foreign antigens could trigger strong TCR signals due to lack of negative selection, and the strong signal could induce a high level of ICOS/PI3K activation that is detrimental to Treg suppressive activity and stability (12, 45, 62). In addition to TCR signaling, proinflammatory cytokines induced by infection such as IL-6, IL-12, or IL-4 can also have a disruptive effect on Treg stability (50, 51, 63). Thus, those Tregs, which respond vigorously are prone to losing their suppressive function, program into an immune-boosting cell, and contribute to clearance of pathogens. Indeed, loss of Treg stability has been observed in many pathologic conditions in response to foreign antigens such as infection and allergy (64–66). This hypothesis would explain many general phenomena in which Tregs can control weak immune responses, while being relatively incompetent to suppress strong immune responses, such as allo-skin grafts. Consistent with this notion, Amigorena et al. have demonstrated that Tregs could down-regulate low-avidity CD8 reactions but promote the high-avidity of CD8^+^ T cell responses to foreign antigen (67).

## Conclusions

Although many controversies remain, more and more pieces of evidence have supported the notion that Tregs can lose Foxp3 expression under certain conditions. In the past 10 years, the extracellular and intracellular signals that maintain or destabilize Foxp3 have been intensively investigated, however, the physiologic function of Treg instability is not fully understood. We propose a hypothesis that Foxp3 instability helps tTreg to distinguish self and non-self-antigens. A better understanding of this question might have important therapeutic applications in a variety of diseases ranging from tumor to infectious disease.

## Author Contributions

ZZ and XZ wrote the manuscript.

### Conflict of Interest Statement

The authors declare that the research was conducted in the absence of any commercial or financial relationships that could be construed as a potential conflict of interest.
